# Global trends in the research on Legg–Calve–Perthes disease in Web of Science

**DOI:** 10.3389/fped.2024.1335118

**Published:** 2024-03-07

**Authors:** Wenbao Qin, Mingwei He, Hongsheng Qin, Qingjun Wei, Haiwei Yan

**Affiliations:** ^1^ Department of Orthopaedics, Hechi Third People’s Hospital, Hechi, Guangxi, China; ^2^ Department of Orthopaedic Trauma and Hand Surgery, First Affiliated Hospital of Guangxi Medical University, Nanning, Guangxi, China; ^3^ Department of Orthopaedics, Fourth Affiliated Hospital of Guangxi Medical University, Liuzhou Worker’s Hospital, Liuzhou, Guangxi, China

**Keywords:** global trends, Legg–Calve–Perthes disease, Web of Science Core Collection, bibliometric analysis, publications

## Abstract

**Background:**

Legg–Calve–Perthes disease (LCPD) is a form of idiopathic femoral head necrosis that can lead to permanent femoral head deformities and premature osteoarthritis in children under the age of 15. Its pathogenesis is utterly and remains to be clarified. Although many research publications on LCPD have emerged during the last few decades, few systematic bibliometric analyses of these articles have been reported.

**Methods:**

A bibliometric analysis was performed to investigate the development processes and hotspots, as well as the collaboration and influence among countries, institutions, authors, journals, and keywords of papers relevant to LCPD from the Web of Science Core Collection (WoSCC) during the period from 1 January 2000 to 30 June 2023.

**Results:**

A total of 2,205 researchers from 916 institutions across 53 countries/regions have contributed to 673 papers published in 199 academic journals. The research on LCPD has shown significant fluctuations but a gradual increase in the number of articles published over the last two decades. The United States leads in the number of publications of LCPD, with the Texas Scottish Rite Hospital for Children being the most productive institution. English, as the most widely used language in the world, was undoubtedly the most popular language. *Herring JA*, who acted as both the corresponding and first author, has contributed to the most co-cited papers published. The most number of LCPD papers are published in the *Journal of Pediatric Orthopaedics*, whereas the *Journal of Bone and Joint Surgery American Volume* garnered the highest total citations, indicating the great importance of these two journals in the field of orthopedics. The most frequently used keywords in published articles were related to the symptoms, mechanisms, and prognosis, revealing the research focus of most scholars.

**Conclusion:**

Our research described the development trends and hotspots in the research field of LCPD and will help researchers make better decisions.

## Introduction

Legg–Calve–Perthes disease (LCPD) is a complex condition affecting the capital femoral epiphysis of the femoral head in children (1). The incidence of LCPD can vary from 0.4 to 29.0 children per 100,000 children under the age of 15 years, with the peak incidence in children aged 4–8 years (2–4). Although it has been widely studied, the etiology of LCPD remains unknown. Evidence showed that it may involve various factors, including genes associated with coagulation and fibrinolysis, proinflammatory factors, and vasoactive substances (5, 6). If appropriate measures are not taken at an early stage, developing degenerative changes associated with arthritis may eventually lead to total hip arthroplasty. Thus, a quantitative analysis of the current states, focal areas, and prospects for LCPD is necessary.

Bibliometrics is an interdisciplinary discipline that could statistically and mathematically analyze publications, including books and periodicals (7). Increasing evidence suggests that bibliometrics has greatly contributed to research trends in medical domains, such as cardiovascular diseases (8), gastrointestinal diseases (9), and diabetes (10). In addition, it can provide reliable data that can be used to expose the research frontier as a handy technique. Although research on LCPD has boomed in recent years, few systematic analyses of these articles using bibliometric analysis have been reported. Hence, the current research aimed to systematically evaluate the international publication productivity of LCPD research, analyze the trends and hotspots, as well as the collaboration and influence among countries, institutions, authors, journals, and keywords of LCPD using the Web of Science Core Collection (WoSCC) from 1 January 2000 to 30 June 2023.

## Methods

### Data source

Learning from previous research strategies, we selected the Science Citation Index Expanded (SCI-Expanded) within the WoSCC database as the data source for this study (11–13). Considering the rapid updation of the database and the avoidance of bias as much as possible, the literature search was completed in 1 day (11 August 2023). To identify all publications, we used “Legg–Calve–Perthes disease” as the TOPIC. A total of 673 relevant publications were finally obtained.

### Bibliometric analysis

The exported publications in a plain text format with “full record and cited references” were named as the file “lcpd_xxx.txt.” and imported to VOSviewer 1.6.19 for further analysis. The Bibliometrix package in R 4.3.1 was used for scientific knowledge map visualization. In VOSviewer, we set the normalization method to association strength and set the minimum thresholds for countries, institutions, authors, keywords, and journals to default value. In addition, GraphPad Prism 8.0 and Adobe Illustrator 15.0.0 were used to generate the figures.

## Results

### Overview of LCPD publications

**Figure 1:**
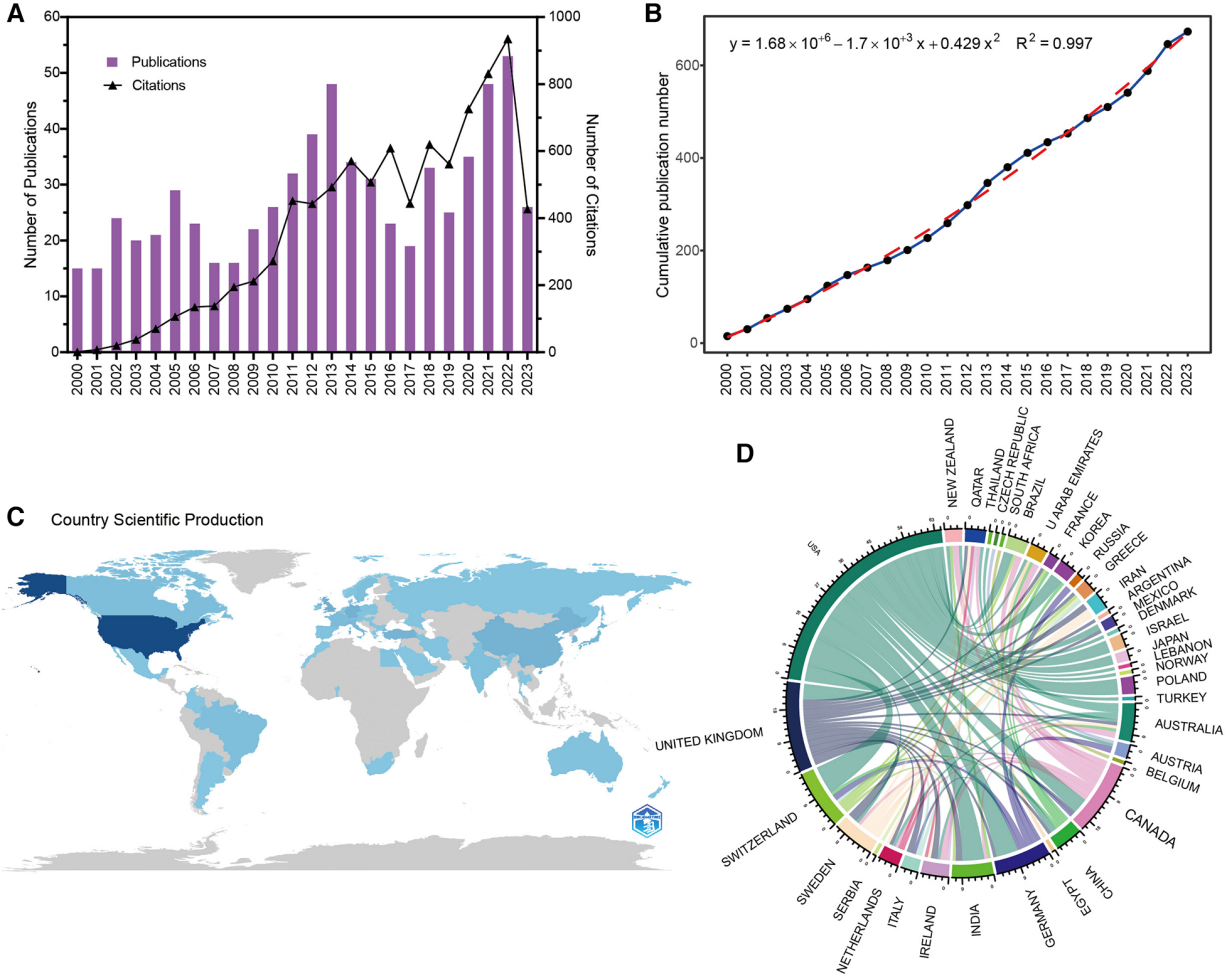
Overview of LCPD publications. (**A**) Number of annual research publications and growth trends on the associations of LCPD from 2000 to 2023. (**B**) Curve fitting of publications’ overall yearly growth trend. (**C**) Geographical distribution of publications on LCPD research from 2000 to 2023. (**D**) Corelationships between different countries on the publication of LCPD.

A total of 673 publications, mainly including 587 articles and 33 reviews, were retrieved from WoSCC (Table 1). Figure 1A depicts the yearly number of publications related to LCPD, which increased from 15 in 2000 to 26 in the first half of 2023, peaking at 53 in 2022. The total citation number of the retrieved articles was 8,813, which increased from 1 in 2000 to 427 in the first half of 2023, peaking at 935 in 2022; the average citation number per article was 13.21. The H-index for these publications was 42, according to data from the website. The polynomial fitting curve of the annual trend of publications is shown in Figure 1B. Although there were large fluctuations over the years, the overall trend showed that the total number of articles published increased, with a correlation coefficient of *R*^2^ = 0.997. These data indicate that the research on LCPD has become the focus of attention and has made rapid progress. Figure 1C depicts the geographical distribution of all papers on LCPD research across all countries and regions. As the figure shows the United States emerged as the country with the highest number of published papers, which not only demonstrates the high productivity of the United States but also shows its significant importance to the study of LCPD. Figure 1D shows the corelationships between different countries in the context of LCPD. It shows that each country has a corelationship with other countries, and especially, the United States, which occupies the most published papers, cooperates closely with every other country.

**Table 1:** Categories of PLCD publications.

Rank	Categories	No.
1	Article	587
2	Review	33
3	Conference abstract	22
4	Editorial material	21
5	Proceedings	10
6	Online publication	7
7	Revision	6
8	Letter	4

No., number of publications.

### Contributions of countries/regions

**Figure 2:**
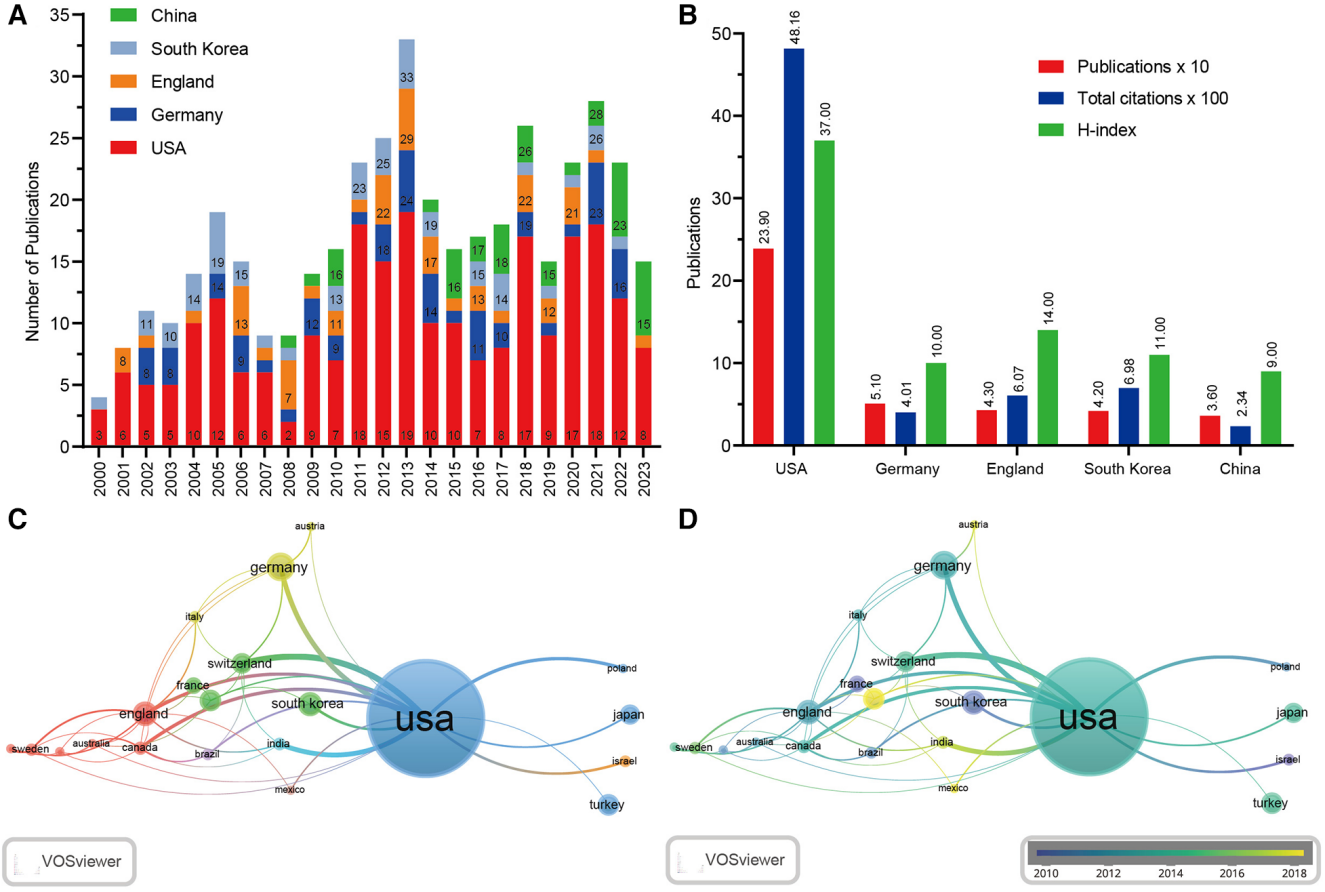
Cooperative relationships between different countries and regions. (**A**) Time histogram of LCPD from the top five high-output countries and regions. (**B**) Citation reports for the top five countries and regions. (**C**) Network of different countries’ visualization. Each node represents one country. The circle size is based on the number of publications. Connecting lines represent collaboration between countries. Different colors represent different items. (**D**) Distribution of countries according to the average time of occurrence. Green and blue circles indicate more past publications, and yellow circles indicate more recent publications.

A total of 53 regions were included in our data (Supplementary Table 1). Figure 2A depicts the top five high-output countries and regions for all writers in the last two decades. It indicates that the articles from the United States largely determined the trend of global publications. Figure 2B shows the United States published the most papers (239/35.51%), followed by Germany (51/7.58%), England (43/6.38%), South Korea (42/6.24%), and China (5.35/36%). Publications from the United States were cited 4,816 times, accounting for 54.61% of the total citations, followed by South Korea (698) and England (607). Moreover, the H-index of the United States was the highest (37), followed by England (14) and South Korea (11). Interestingly, compared to Germany, South Korea had a relatively lower number of papers but a much higher citation count and H-index, suggesting that South Korea has a more influential impact in the field of LCPD (Table 2). Figure 2C shows that the United States was the main continent for LCPD research, collaborating closely with many countries, including Canada, Japan, England, and China. Figure 2D presents the distributions of the countries according to the average occurrence time, with purple indicating the earlier, while yellow indicating the later. As we can see in the chart, the United States, Germany, South Korea, and France were among the countries that published earlier, while India, Mexico, and Australia were among the countries that published more recently, indicating that LCPD research is gradually receiving attention from countries around the world.

**Table 2:** Top five countries/regions ranked by the number of publications.

Rank	Countries/regions	No.	Count (%)	TC	H-index
1	United States	239	35.51	4,816	37
2	Germany	51	7.58	401	10
3	England	43	6.39	607	14
4	South Korea	42	6.24	698	11
5	China	36	5.35	234	9

No., number of publications; TC, total citations.

### Leading productive institutions

**Figure 3:**
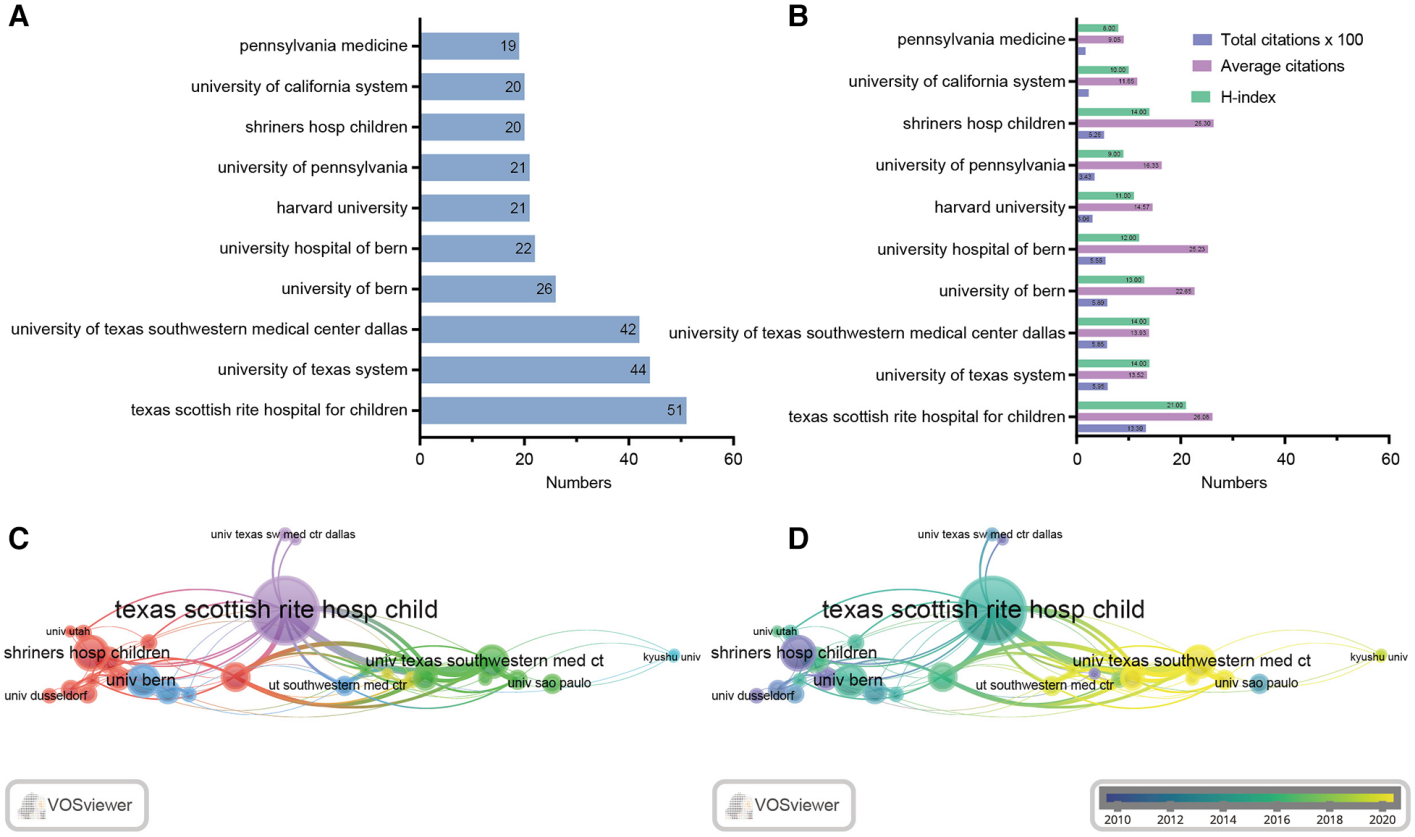
Most productive institutions and their collaborations. (**A**) Top 10 institutions involved in research on LCPD. (**B**) Distribution of citations (×100), citation frequency per paper, and H-index of the top 10 institutions. (**C**) Network of different institutions’ visualization. Each node represents one institution. The circle size is based on the number of publications. Connecting lines represent collaboration between countries. Different colors represent different items. (**D**) Distribution of institutions according to the average time of occurrence. Green and blue circles indicate more past publications, and yellow circles indicate more recent publications.

In our data, 916 institutions involved in LCPD were detected (Supplementary Table 2). Figure 3A lists the top 10 productive institutions. Specifically, Texas Scottish Rite Hospital for Children has 51 papers, followed by the University of Texas System (44) and the University of Texas Southwestern Medical Center Dallas (42). Interestingly, we found that only 2 of these top 10 most productive institutions, the University of Bern and the University Hospital of Bern, were from Switzerland, while the remaining 8 institutions were all from the United States. This result once again highlights the significance of articles from the United States in determining the global publication trends. As shown in Figure 3B and Table 3, the 51 publications from Texas Scottish Rite Hospital for Children in the United States ranked first with 2,608 citations (51.13 citations per article), and the H-index was 21. It is worth noting that in terms of the quality of articles, the H-index values of these 10 institutions differ slightly, which suggests there was no significant gap between institutions. In addition, VOS viewer analysis was also performed to detect the relationships among these institutions. Figure 3C shows that the institutions were divided into four clusters, with Texas Scottish Rite Hospital for Children collaborating closely with many other institutions. Figure 3D presents the distribution of the institutions based on the mean occurrence time, where blue indicates earlier publication and yellow indicates later publication. As we can learn from this figure, Shriners Hosp Children was the institution with the largest number of publications on LCPD in earlier times, while the University of Texas Southwestern Medical Center Dallas was more recent.

**Table 3:** Top 10 institutions ranked by the number of publications.

Rank	Institutions	No.	Count (%)	TC	AC	H-index
1	Texas Scottish Rite Hospital for Children	51	7.58	1,330	26.08	21
2	University of Texas System	44	6.54	595	13.52	14
3	University of Texas Southwestern Medical Center Dallas	42	6.24	585	13.93	14
4	University of Bern	26	3.86	589	22.65	13
5	University Hospital of Bern	22	3.27	555	25.23	12
6	Harvard University	21	3.12	306	14.57	11
7	University of Pennsylvania	21	3.12	343	16.33	9
8	Shriners Hosp Children	20	2.97	526	26.3	14
9	University of California System	20	2.97	233	11.65	10
10	Pennsylvania Medicine	19	2.82	172	9.05	8

No., number of publications; TC, total citations; AC, average citation.

### Language distributions

**Figure 4:**
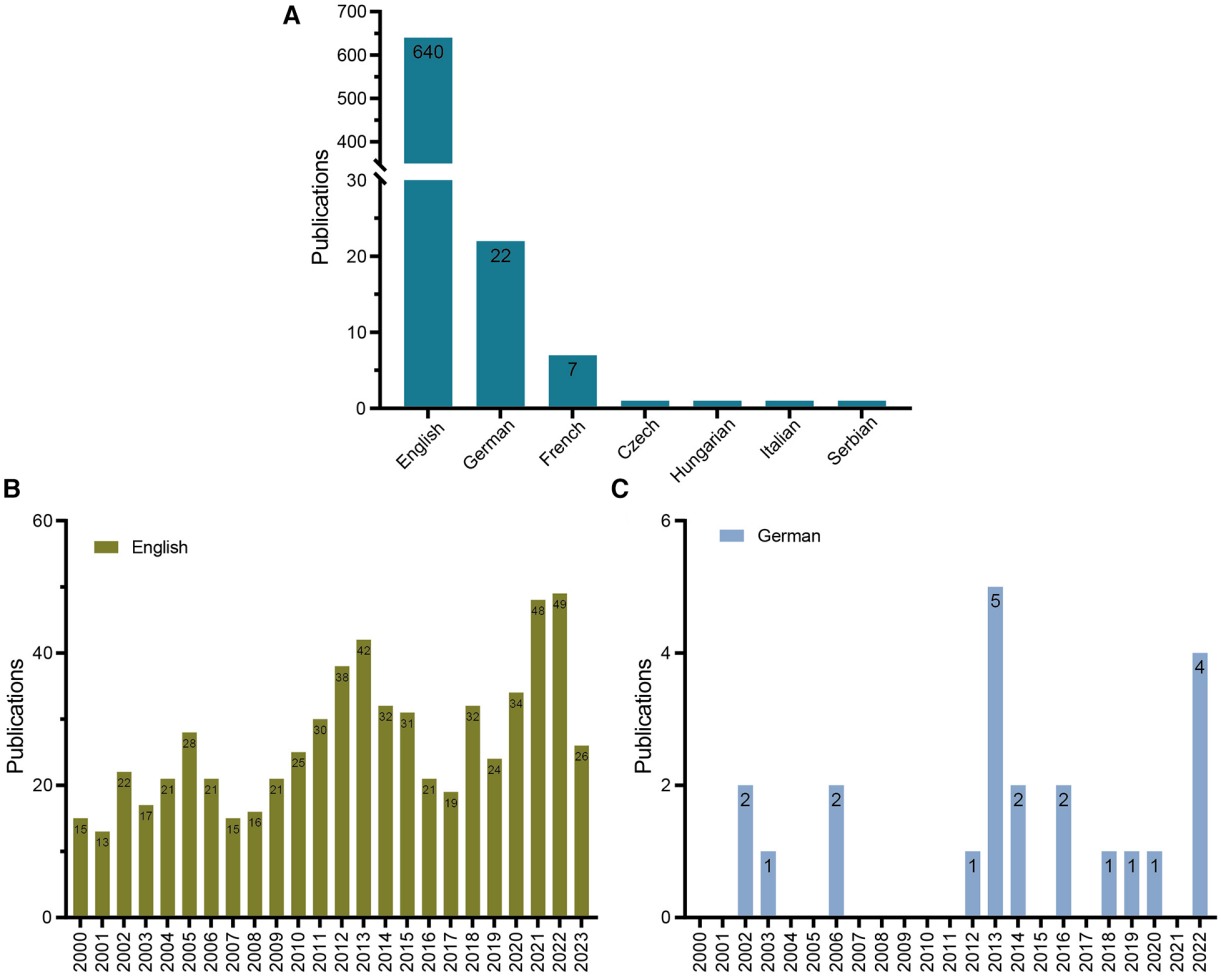
Most productive languages in LCPD. (**A**) Most published language for research in LCPD. (**B**) Annual global number of LCPD publications published in English. (**C**) Annual global number of LCPD publications published in German.

Seven languages related to publications of LCPD were listed in this research (Figure 4A). As expected, English ranked first with 640 publications, followed by German with 22. French ranked third with seven publications. In addition, we found a fluctuating but gradually increasing trend in English publications from 15 in 2000 to 49 in 2022 (Figure 4B). The number of German publications (second), however, did not show an increasing trend (Figure 4C). This reason may be related to the gradual development of English as a global language in the past 20 years.

### Distribution of authors

**Figure 5:**
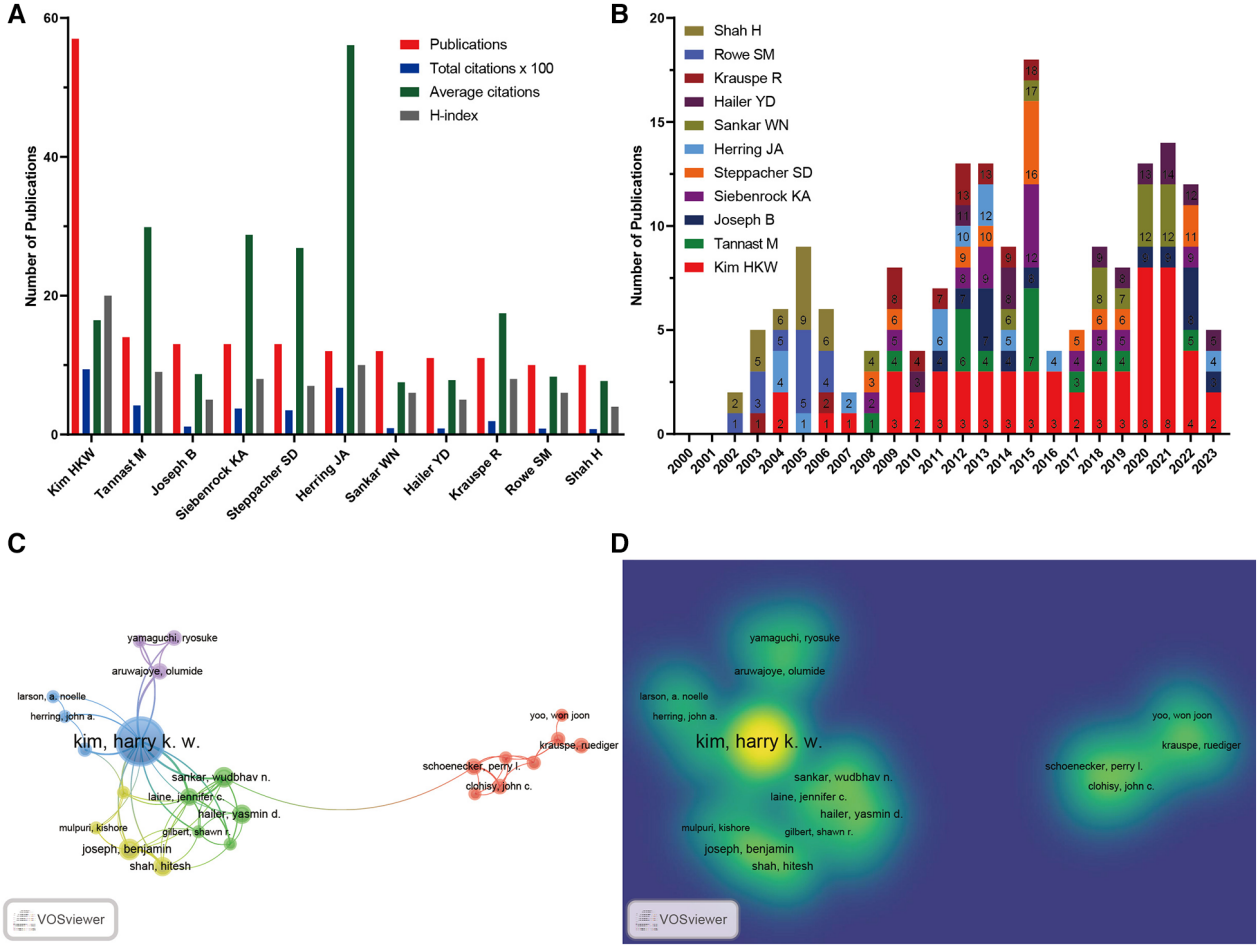
Most productive authors and their collaborations. (**A**) Distribution of publications, total citations (×100), citation frequency per paper, and H-index of the top 10 authors. (**B**) Time histogram of LCPD from the top 10 high-output authors. (**C**) Network of different authors’ visualization. Each node represents one author. The circle size is based on the number of publications. Connecting lines represent collaboration between authors. Different colors represent different items. (**D**) Density map showing the heatmap of authors during the period from 1 January 2000 to 30 June 2023.

A total of 2,205 researchers associated with the LCPD publications were identified in our data (Supplementary Table 3). Ranking by the number of papers, the top 10 most productive authors were *Kim HKW* (57, the United States), *Tannast M* (14, Switzerland), *Joseph B* (13, India), *Siebenrock KA* (13, Switzerland), *Steppacher SD* (13, Switzerland), *Herring JA* (12, the United States), *Sankar WN* (12, the United States), *Hailer YD* (11, Sweden), *Krauspe R* (11, Deutschland), *Rowe SM* (10, South Korea), and *Shah H* (10, India) (Figure 5A and Table 4). We found that the most productive paper-published author, *Kim HKW*, works at Scottish Rite Children (2222 Welborn St, Dallas, TX, the United States), which may explain why Texas Scottish Rite Hospital for Children and the United States were the most productive paper-published institution and region, respectively (Figures 3A,B). The H-index of *Kim HKW* was 20, which means that 20 papers have each been cited at least 20 times in his published papers (Figure 5B). These results indicate *Kim HKW* is the most productive author in the field of LCPD. In addition, we found that *Herring JA* ranked sixth in terms of the number of publications; however, his average citation was the highest, suggesting that *Herring JA* is the most influential author in the field of LCPD. We further detected the top 10 most cited papers related to LCPD and found that two papers of *Herring JA* ranked first and third, both published in 2004 (Table 5). Furthermore, to detect the relationships among these authors, VOSviewer analysis was also performed in our research. Figures 5C,D demonstrate that the authors were divided into three clusters, with *Kim HKW*, as the most influential author, collaborating closely with many other authors.

**Table 4:** Top 10 authors ranked by the number of publications.

Rank	Institutions	No.	Count (%)	TC	AC	H-index
1	Kim HKW	57	8.47	938	16.46	20
2	Tannast M	14	2.08	418	29.86	9
3	Joseph B	13	1.93	113	8.69	5
4	Siebenrock KA	13	1.93	374	28.77	8
5	Steppacher SD	13	1.93	349	26.85	7
6	Herring JA	12	1.78	673	56.08	10
7	Sankar WN	12	1.78	90	7.5	6
8	Hailer YD	11	1.63	86	7.82	5
9	Krauspe R	11	1.63	192	17.45	8
10	Rowe SM	10	1.49	83	8.3	6
10	Shah H	10	1.49	77	7.7	4

No., number of publications; TC, total citations, AC, average citation.

**Table 5:** Top 10 papers with the highest citations.

Rank	Title	CA	Journal	Year	TC	IF
1	Legg–Calve–Perthes disease. Part II: Prospective multicenter study of the effect of treatment on outcome	Herring JA	*Journal of Bone and Joint Surgery American Volume*	2004	231	5.3
2	Arthroscopic capsular plication and labral preservation in borderline hip dysplasia two-year clinical outcomes of a surgical approach to a challenging problem	Domb BG	*American Journal of Sports Medicine*	2013	219	4.8
3	Legg–Calve–Perthes disease. Part I: Classification of radiographs with use of the modified lateral pillar and Stulberg classifications	Herring JA	*Journal of Bone and Joint Surgery American Volume*	2004	182	5.3
4	Breed susceptibility for developmental orthopedic diseases in dogs	LaFond E	*Journal of The American Animal Hospital Association*	2002	171	1.3
5	The prevalence of acetabular retroversion among various disorders of the hip	Ezoe, M	*Journal of Bone and Joint Surgery American Volume*	2006	139	5.3
6	The relationship between diagnosis and outcome in arthroscopy of the hip	Vail, TP	*Arthroscopy—The Journal of Arthroscopic and Related Surgery*	2001	124	4.7
7	Femoral morphology differs between deficient and excessive acetabular coverage	Tannast, M	*Clinical Orthopaedics and Related Research*	2008	115	4.2
8	Osteonecrosis of the femoral head of laboratory animals: The lessons learned from a comparative study of osteonecrosis in man and experimental animals	Boss, JH	*Veterinary Pathology*	2003	101	2.4
9	Pathophysiology and new strategies for the treatment of Legg–Calve–Perthes disease	Kim, HK Kim, HK	*Journal of Bone and Joint Surgery American Volume*	2012	86	5.3
10	Ibandronate for prevention of femoral head deformity after ischemic necrosis of the capital femoral epiphysis in immature pigs		*Journal of Bone and Joint Surgery American Volume*	2005	86	5.3

CA, corresponding authors; TC, total citations; IF, impact factor 2023.

### Top 10 most cited papers

The top 10 most cited papers are listed in Table 5. The most frequent one was a prospective multicenter study we mentioned earlier, “*Legg–Calve–Perthes disease. Part II: Prospective multicenter study of the effect of treatment on outcome*” by *Herring JA* et al. (14) in 2004, which was cited 231 times. The researchers enrolled 438 cases of 451 hip Perthes disease from 38 institutions in the United States. They found that Stulberg grading evaluation after bone maturation showed that grade I or II was excellent, and the incidence rate of patients younger than 8 years old was significantly higher than that of patients older than 8 years old. The second most cited paper was “*Arthroscopic Capsular Plication and Labral Preservation in Borderline Hip Dysplasia Two-Year Clinical Outcomes of a Surgical Approach to a Challenging Problem*” by *Domb BG* et al. (15) in 2013, which was cited 219 times so far. The authors found that the arthroscopic approach was beneficial to patients with borderline dysplasia. The third paper was “*Legg–Calve–Perthes disease. Part I: Classification of radiographs with use of the modified lateral pillar and Stulberg classifications*” by *Herring JA* et al. (16) in 2004, which was cited 182 times. In this study, *Herring* proposed to improve the lateral column classification on the basis of the original classification (17), which was published in his paper “*The lateral pillar classification of Legg–Calvé–Perthes disease*” in 1992, namely, to add B/C type between the original B type and C type; the results indicated that the modified lateral pillar classification and the redefined Stulberg classification were sufficiently reliable and accurate for use in studies of LCPD.

### Journal distributions

**Figure 6:**
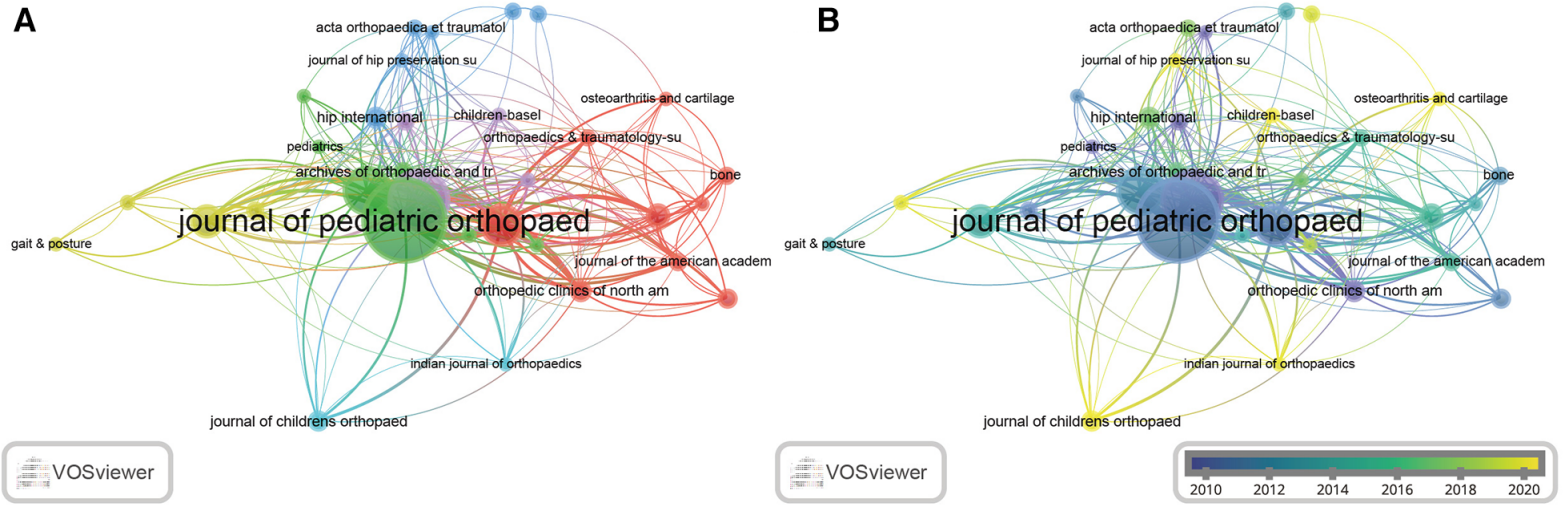
Coauthorship map of high-yielding journals. (**A**) In the visualization map, one node represents a journal, and its size is proportional to the number of publications. Different colors represent different items. (**B**) Distribution of journals according to the average time of occurrence. Green and blue circles indicate more earlier publications, and yellow circles indicate more recent publications.

A total of 199 journals were detected in this study (Supplementary Table 4). We further identified the top 10 journals with the most numbers of publications related to LCPD (Table 6). The results showed that the *Journal of Pediatric Orthopaedics* (IF = 2.5372, 2021) publishes the most number of LCPD papers with 89, followed by the *Journal of Pediatric Orthopaedics Part B* (IF = 1.4730, 2021) with 56, *Clinical Orthopaedics and Related Research* (IF = 4.8371, 2021) with 40, the *Journal of Bone and Joint Surgery American Volume* (IF = 6.5581, 2021) with 32, and *International Orthopaedics with 22* (IF = 3.4793, 2021) (Table 6)*.* Interestingly, the *Journal of Pediatric Orthopaedics* has the greatest number of publications related to LCPD, but the citation count only ranks second with 1,093. On the contrary, the *Journal of Bone and Joint Surgery American Volume* ranks fourth according to the number of publications, but it ranks first in the number of citations with 1,351. We also detected the quartile in the category of these journals according to the Journal Citation Report (JCR) 2023. The results showed that *Clinical Orthopaedics and Related Research* and the *Journal of Bone and Joint Surgery American Volume* were classified in Q1, while *International Orthopaedics*, the *Journal of Orthopaedic Research*, *Orthopedic Clinics of North America*, *Archives of Orthopaedic and Trauma Surgery*, and *Pediatric Radiology* were classified in Q2. The most productive journal, the *Journal of Pediatric Orthopaedics*, was classified in Q3, as well as *Hip International* and the *Journal of Children’s Orthopaedics*. Only one journal, the *Journal of Pediatric Orthopaedics Part B*, was classified in Q4. In addition, VOSviewer analysis was also performed to detect the distributions of LCPD publications in our research. The result showed that the *Journal of Pediatric Orthopaedics* occupies the most cooperation number with other journals (Figure 6A) and is one of the earliest journals involved in LCPD (Figure 6B). It is worth noting that the *Journal of Pediatric Orthopaedics* is a leading journal that mainly focuses specifically on traumatic injuries, according to its website. In addition, there were some new journals, according to the average time of occurrence in Figure 6B, such as the *Journal of Children’s Orthopaed* (IF = 1.9168, 2021), *Children-Basel* (IF = 2.8350, 2021), and *Osteoarthritis and Cartilage* (IF = 7.5065, 2021).

**Table 6:** Top 10 journals with the highest numbers of publications.

Rank	Journal	No.	TC	JCR	IF
1	*Journal of Pediatric Orthopaedics*	89	1,093	Q3	1.7
2	*Journal of Pediatric Orthopaedics Part B*	56	445	Q4	1.1
3	*Clinical Orthopaedics and Related Research*	40	921	Q1	4.2
4	*Journal of Bone and Joint Surgery American Volume*	32	1,351	Q1	5.3
5	*International Orthopaedics*	22	279	Q2	2.7
6	*Journal of Orthopaedic Research*	13	176	Q2	2.8
7	*Hip International*	11	89	Q3	1.5
7	*Orthopedic Clinics of North America*	11	239	Q2	1.8
9	*Journal of Children’s Orthopaedics*	10	38	Q3	1.4
10	*Archives of Orthopaedic and Trauma Surgery*	9	55	Q2	2.3
10	*Pediatric Radiology*	9	153	Q2	2.3

No., number of publications; TC, total citations; JCR, journal citation reports 2023; IF, impact factor 2023.

### Keyword distributions

**Figure 7:**
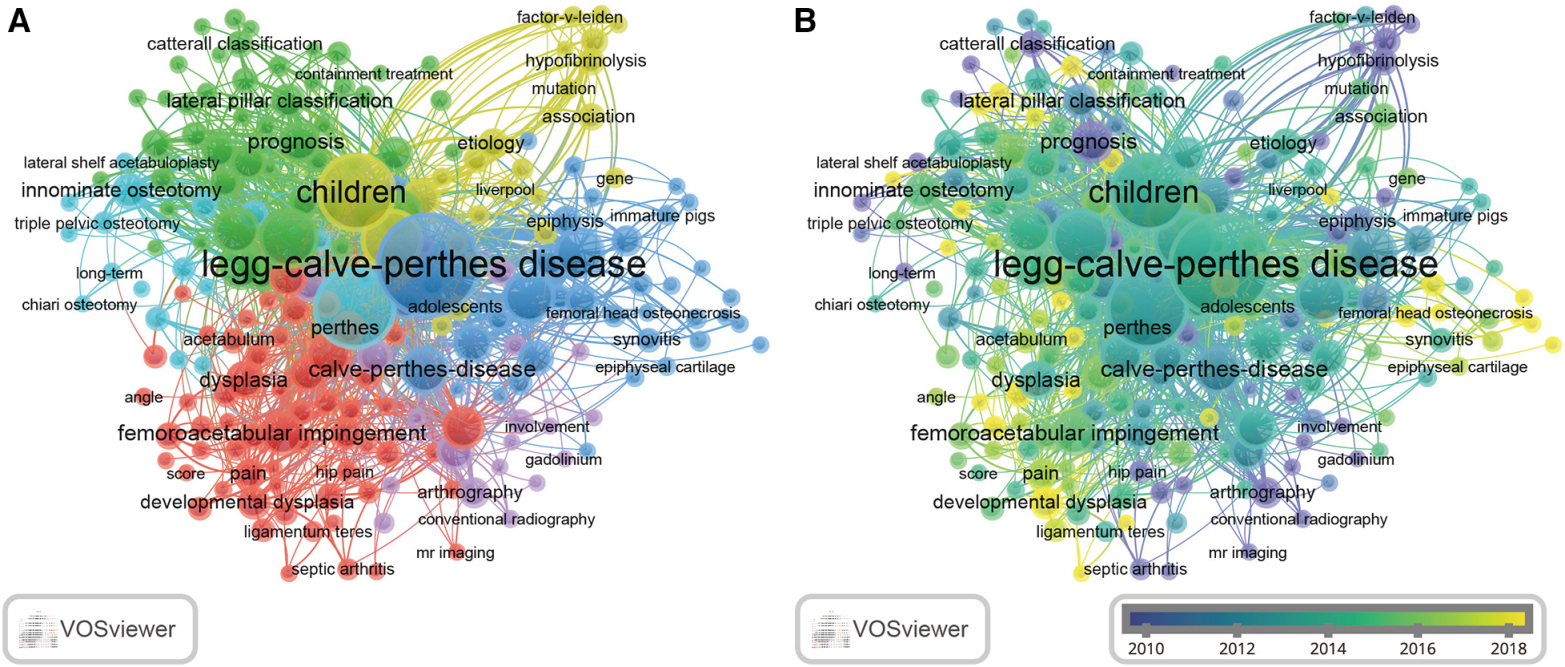
Co-occurrence network of keywords. (**A**) Lines between nodes represent co-occurrence between different keywords. Each node represents one keyword. The circle size is based on the number of occurrences. Different colors represent different items. (**B**) Distribution of keywords according to the average time of occurrence. Green and blue circles indicate more earlier publications, and yellow circles indicate more recent publications.

We further performed VOSviewer analysis of the co-occurring keywords. The result indicated that the keywords could mainly be divided into six categories, including “symptom (red),” “imageology (purple),” “mechanism (dark blue),” “etiology (yellow),” “prognosis (green),” and “treatment (light blue)” (Figure 7A). In the “symptom” group, the most popular keywords were “femoroacetabular impingement,” “dysplasia,” and “pain.” These keywords accurately describe the typical characteristics of LCPD. In the “imageology” group, the most popular keywords were “MRI” and “arthrography,” which represent the popular detection methods currently. In the “mechanism” group, “Legg–Calve–Perthes disease” was the most frequent in many papers (Figure 7A) and appeared to be one of the earliest keywords, also (Figure 7B). Moreover, we noticed that “epiphysis,” “angiogenesis,” and “interleukin-6” had the most occurrences in the “mechanism” group. It is worth noting that “interleukin-6” was a new keyword appearing in LCPD recent year (Figure 7B). Li et al. (18) reported that LCPD patients usually have a high level of interleukin-6 in their plasma, and the high concentration of interleukin-6 can prompt human umbilical vein endothelial cells to secrete CD31^+^/CD42b^−^ endothelial microparticles, which in turn promote endothelial cell apoptosis and endothelial dysfunction and inhibit angiogenesis. In the “etiology” group, the most popular keywords were “children,” “epidemiology,” and “mutation,” indicating that the research trend of LCPD is moving toward mutations in children. In the “prognosis” group, we noticed “classification” and “prognosis,” suggesting that the outcome was associated with the disease classification. In the “treatment” group, “osteotomy” was the most prevalent keyword. Evidence indicated that osteotomy shows improved clinical and radiological outcomes in children with LCPD (19–21).

## Discussion

This was the first bibliometrics analysis on LCPD worldwide, aiming to detect the trends and research hotspots of LCPD based on the WoSCC database. Herein, 673 papers were retrieved from 1 January 2000 to 30 June 2023. According to the polynomial fitting curve (Figure 1), although the number of published papers fluctuated less over the two decades, the overall trend was toward more and more published papers. This also indicates that more and more researchers are interested in the topic of LCPD.

Publications were distributed all over the world, but productivity was modest in many places. In this dataset, the United States rated first in the number of publications among the top 10 countries and regions, indicating a very productive country in LCPD research (Figure 2). Among the top 10 institutions and authors in the field of LCPD research, the United States has 8 institutions and 3 scholars, indicating that the United States has the most outstanding institutions and expert scholars, which helps explain why the United States has had such an impact on the topic over the past 20 years. Moreover, the United States has the highest H-index among these countries. It may be because researchers in the United States first proposed the treatment and care for LCPD (14, 16). In addition, the United States has been more intensively studying this issue than other countries around the globe. This suggests that Chinese scholars and institutions in this field should improve the quality of their work.

Institutions are important places for research. In this research, we found that almost all of the top 10 institutions ranking with the most publications came from the United States, demonstrating its strong scholarly competence in this field (Figure 3). *Kim HKW*, *Tannast M*, and *Joseph B* were the top three scholars in the research on LCPD based on the number of publications (Figure 5). Therefore, to keep up with the latest developments in this field, we should pay more attention to their work and give it higher priority. Texas Scottish Rite Hospital for Children was the most productive institution, with 51 published articles and 1,330 citations, followed by the University of Texas System (44 published articles, 595 citations) and the University of Texas Southwestern Medical Center Dallas (42 published articles, 585 citations). Interestingly, *Kim HKW*, who is affiliated with the University of Texas System, has authored the majority of the papers. The highest cited article of *Kim HKW* was a review published in 2012, which introduced the pathophysiology and new strategies for the treatment of LCPD (86 citations) (22) and provided clinicians with strategies for the treatment and prevention of LCPD. It is worth noting that *Kim HKW*'s latest study aimed at measuring the brace adherence of the A-frame, an equipment used to contain the deformed femoral head and improve femoral head remodeling in patients with LCPD using temperature sensors, and to identify factors that influence adherence. The study found that the compliance of A-frame braces was significantly associated with age at the time of treatment, previous Petrie casting, and daily prescribed brace wear (23). This latest study provides new insights and suggestions for the treatment and prevention of LCPD.

Among the top 10 most cited articles, studies on human LCPD treatment accounted for 90% (9/10), while only one paper was related to dogs, indicating that research on human LCPD treatment is a hot topic of interest for most scholars (Table 5). Three scholars whose articles have been cited over 150 times were *Herring JA*, *Domb BG*, and *LaFond E*. *Herring JA*'s article, published in 2004, occupied the highest number of citations and ranked first. The research illustrated that Stulberg grading evaluation after bone maturation showed that grade I or II was excellent, and the incidence rate of patients younger than 8 years old was significantly higher than that of patients older than 8 years old. Therefore, the highest citation count indicates that other scholars have given his work a high degree of recognition (14). What is more, *Domb* et al. (24) presented acetabular dysplasia as a developmental malformation that results in poor coverage of the femoral head by the acetabulum, a tendency for subluxation or dislocation of the joint, shallow acetabulum, and outward centrality of the hip joint in this study. In addition, the studies of *LaFond* et al. (25) served to increase the impact of veterinarians on developmental straightening disease and promote control of this disease by identifying breeds that may benefit from environmental interventions such as breeding programs or ecological interventions. It is worth noting that *LaFond E*, a veterinarian, authored the only paper related to animals, ranking No.4 of the top 10 most cited papers, indicating that his work was highly acknowledged by other scholars.

The view of keywords (Figures 7A,B) revealed that the hot topic people have always been concerned about all the time was “Legg–Calve–Perthes disease.” Based on the clustering, we found that most of the research studies focused on the “symptom (red),” “mechanism (dark blue),” and “prognosis (green)” studies of LCPD. From this perspective, it can be seen that in the last two decades, scholars have attached great importance to investigating the causes and mechanisms of LCPD.

It also implies that most research on LCPD has focused on its causes, mechanisms, and treatment methods. As far as we know, the mechanism of LCPD remains to be fully detailed. An article details that LCPD patients usually have a high level of interleukin-6 in their plasma, and the high concentration of interleukin-6 can prompt human umbilical vein endothelial cells to secrete CD31^+^/CD42b^−^ endothelial microparticles, which lead to endothelial cell apoptosis and dysfunction, as well as inhibit angiogenesis (18). Thus, we can conduct more research on interleukin-6 to unveil the cause of LCPD in the future.

Notably, the top 10 most prolific journals exhibit a modestly low IF, which is one of the important indexes to evaluate the influence of SCI journals (Table 6). This indicates that publishing research on LCPD in high-quality journals remains challenging. Among these journals, the *Journal of Bone and Joint Surgery American Volume* has the highest IF. In contrast, the IF of other journals ranges only from 1 to 3. The *Journal of Bone and Joint Surgery American Volume*, namely, *JBJS*, belongs to the top journals in orthopedic and publishes evidence-based research to improve the quality of care for orthopedic patients. *JBJS*, the journal committed to providing the best research data in the world, has been the most respected source of information for orthopedic surgeons and researchers for over 100 years and is the gold standard for peer-reviewed research information in the field of orthopedics. Thus, although the number of publications in *JBJS* was not the highest, the total citations indicate its significant impact. *Clinical Orthopaedics and Related Research*, namely, CORR, ranking second in IF among these 10 journals, is committed to disseminating new and important orthopedic knowledge and is a leading peer-reviewed orthopedic journal and a publication of the Society of Bone and Joint Surgeons. CORR brings readers the latest clinical and basic research as well as informed opinions that shape plastic surgery practice today, thus providing an opportunity to practice evidence-based medicine. In addition, we also noticed that the *Journal of Pediatric Orthopaedics*, which has an IF lower than 3, publishes the most number of papers; however, the citations were not the most. These results indicate that a journal with a large number of publications does not necessarily have a large impact.

This study conducted a comprehensive visual analysis of LCPD in a clear and objective manner. However, there were some limitations that need to be considered. First, this analysis only relied on the WoSCC database and ignores other databases, such as PubMed and Scopus, which may result in a limited extraction of publications. However, the WoSCC database includes influential and high-quality journals, providing a wide range of citation index records, making it suitable for scientific and bibliometric analysis. Second, this study did not include articles earlier than 2000, potentially overlooking several high-quality publications. However, due to the extensive coverage of authoritative and influential academic journals in research, the results and findings remain robust (26).

## Conclusions

This analysis shows that the number of published papers on LCPD research has fluctuated slightly in the last two decades. However, the overall trend indicates that more and more articles are being published. This indicates that the research on LCPD has good research prospects. The United States emerged as a major producer country with more significant influence in this field. Osteotomy remains an important way to improve the clinical outcome in children with LCPD. In recent years, the clinical and molecular biology research related to LCPD has attracted widespread attention, with interleukin-6 becoming a potential hotspot in LCPD research. The research in this article will help scholars better understand the current status of LCPD research from a large perspective.

## Data Availability

The original contributions presented in the study are included in the article/Supplementary Material; further inquiries can be directed to the corresponding authors.

## Supplementary material

Supplementary Table 1 All countries’/regions’ distribution.

Supplementary Table 2 All institutions’ distribution.

Supplementary Table 3 All authors’ distribution.

Supplementary Table 4 All journals’ distribution.
